# SELF-PERCEIVED BARRIERS TO RETURNING TO WORK AMONG EMPLOYEES WITH A LOW EDUCATIONAL LEVEL ON LONG-TERM SICK LEAVE: THE “NOW WHAT” LARGE-SCALE INTERVIEW STUDY

**DOI:** 10.2340/jrm.v57.40604

**Published:** 2025-05-15

**Authors:** Linn V. LERVIK, Elisabeth FROSTESTAD, Kine STRØMSTAD, Ida G. GULLIKSEN, Monica LILLEFJELL, Jens C. SKOGEN, Elin EKBLADH, Randi W. AAS

**Affiliations:** 1 Department of Rehabilitation Science and Health Technology, Faculty of Health Sciences, OsloMet–Oslo Metropolitan University, Oslo, Norway; 2 Department of Public Health, Faculty of Health Sciences, University of Stavanger, Stavanger, Norway; 3 Department of Health Promotion, Norwegian Institute of Public Health, Bergen, Norway; 4 Department of Neuromedicine and Movement Science, Faculty of Medicine and Health Sciences, Norwegian University of Science and Technology, Trondheim, Norway; 5 Centre for Evaluation of Public Health Measures, Norwegian Institute of Public Health, Oslo, Norway; 6 Alcohol and Drug Research Western Norway, Stavanger University Hospital, Stavanger, Norway; 7 Department of Health, Medicine and Caring Sciences, Linköping University, Linköping, Sweden; 8 Institute for Health, Faculty of Health Sciences, VID Specialized University, Stavanger, Norway

**Keywords:** barriers, content analysis, interview, ICF, educational level, NOW WHAT, long-term sick leave, return to work

## Abstract

**Objective:**

Because employees with low educational levels have the highest rates of sick leave, this study aimed to identify the self-perceived return-to-work barriers of employees with low educational levels on long-term sick leave.

**Methods:**

Employees on long-term sick leave with primary/secondary educational attainment were included from the NOW WHAT large-scale interview study (*n *= 122). The World Health Organization’s system of classifying functioning, disability, and health guided the deductive content analysis.

**Results:**

1,942 meaning units describing return-to-work barriers across all classifications were identified. The most frequent components were body functions (*n *= 552, 28%, mean = 4.5), with mental functions (e.g., sleep, tiredness, emotional and cognitive functioning) the most frequent barriers; environmental factors (*n *= 414, 21%, mean = 3.4), with services, systems ,and policies (e.g., social security, healthcare system) the most prevalent barriers; activity limitations (*n *= 352, 18%, mean = 2.9); and personal factors (*n *= 323, 17%, mean = 2.6).

**Conclusion:**

Employees with low educational levels on long-term sick leave described a wide range of return-to-work barriers and combinations thereof. In addition to health-related functional barriers, identifying environmental, activity-limitation, and personal barriers is important to enhance understanding of this group’s potential determinants of absence from work.

## LAY ABSTRACT

Employees with low educational levels on sick leave have the highest sick-leave rates, yet limited research exists on their return-to-work barriers. We explored interviews with employees on long-term sick leave with primary/secondary educational attainment. The World Health Organization’s classification system of function, disability, and health guided the analysis of return-to-work barriers. We identified barriers across all of these components. The most frequent was body functions, within which frequent barriers related to mental functions, such as sleep problems, tiredness, and emotional and cognitive functioning. This was followed by environmental factors, within which the most frequent category was services, systems, and policies, with the barriers primarily concerning social security and the healthcare system. Then followed activity limitations and personal factors components. Our findings indicate a need for stakeholders in the return-to-work process to take a broader perspective of barriers beyond health-related functional barriers to better tailor this group’s needs.

Work is generally considered good for people’s health (1, 2). At the same time, 1 in 4 employees on long-term sick leave do not return to work (3, 4). Long-term sick leave can have both micro- and macro-level consequences and poses global challenges (5). There is no agreed definition of long-term sick leave, yet the term is commonly used for sickness absences of 6 weeks or more (6–8).

Research on sick leave and returning to work (RTW) has traditionally focused on specific diagnostic groups, particularly musculoskeletal disorders (MSDs) (9, 10). However, employees’ RTW processes and their outcomes are complex, influenced by factors beyond a medical diagnosis, such as psychosocial determinants, RTW expectations, and workplace conditions (9, 11). There is a need to identify cross-cutting factors, which are common influences that shape the RTW process regardless of specific disorders (9, 10, 12).

One factor that stands out as being associated with the length of RTW across diagnostic groups is education (9, 10, 12). More specifically, a lower educational level is negatively associated with RTW. This trend is similar across the large diagnostic groups of MSDs (9, 12), common mental disorders (CMDs) (9, 10, 12), cardiovascular diseases (CVDs) (9, 10), and cancer (CA) (10). Several meta-analyses have indicated that individuals with a high educational level are approximately twice as likely to return to work after sick leave as those with a low educational level (13, 14). Research investigating early disability pensions generally supports an educational gradient with a higher risk among those with lower educational levels, and particularly for those who have not completed their education (15–17). Nevertheless, this gradient is seldom used to inform what sick-listed employees in different educational groups require and receive in terms of RTW services. It could be that the types and levels of service needs vary across the educational gradient.

Possible reasons for an educational gradient are related to aspects of work-related psychosocial factors – that is, perceived work strain and motivation, whereby employees with high education often have high-quality jobs with stronger job resources, greater flexibility, and autonomy (10, 12, 18, 19). This is in line with existing theories, such as the job demand–control (–support) model (20, 21) and the illness flexibility model (19). In addition, education contributes to general knowledge and health literacy, which in turn may yield better health behaviours, such as from decisions related to leading a healthier lifestyle (22).

In Norway, over 60% of adults have primary or secondary school education (International Standard Classification of Education [ISCED], levels 1–3), with a sick-leave rate of approximately 7% by the end of 2022. Conversely, under 40% have higher education (ISCED levels 5–8), with a sick-leave rate of approximately 5% (23, 24). The former group is often understudied in work health and RTW research, highlighting the need for more attention to this large demographic (25).

To the best of our knowledge, some qualitative studies have had a cross-cutting focus on RTW barriers regardless of diagnoses; however, none has investigated barriers in relation to educational level (26–29). In the present study, we aim to identify self-perceived RTW barriers among employees with low educational levels on long-term sick leave.

## Methods

### Design

We used material from the NOW WHAT RTW barrier study, a large-scale telephone interview study involving long-term sick-listed employees (*n *= 264) living in 1 county in Norway that includes both large cities and areas of countryside. This design, involving a substantial number of informants, was used to uncover a broad spectrum of experiences to achieve sufficient information power (30–34). Only the interviewed employees with low educational levels (defined as primary and secondary school level, as indicated in in Table I) on long-term sick leave (*n* = 122) were included in the current study.

**Table I:** Overview of participants’ sociodemographic characteristics (*n *= 122)

Characteristics	
Low educational level *n* (%)	
Primary school (levels 1–2)	7 (6)
Secondary school (levels 3–4)	115 (94)
Sex, *n* (%)	
Male	69 (57)
Female	53 (43)
Age, years^a^ mean (SD, range)	47.24 (10.83, 21–68)
Diagnosis group^a^, *n* (%)	
Musculoskeletal disorders	60 (49)
Common mental disorders	26 (21)
Cardiovascular disorders	15 (12)
Others^c^	22 (18)
Residence, *n* (%)	
City	68 (56)
Rural	54 (44)
Employment sector, *n* (%)	
Public	29 (24)
Private	87 (71)
Private, with public duties	6 (5)
Type of work^e^, *n* (%)	
Mining and quarrying	21 (17)
Construction	19 (16)
Transport and storage	17 (14)
Wholesale and retail trade	16 (13)
Human health and social work activities	12 (10)
Education	8 (7)
Public administration and defence	6 (5)
Other	23 (19)
Leader responsibility, *n* (%)	
Yes	37 (30)
Percentage of full-time equivalent^a^, *n* (%)	
≥ 100%	102 (84)
< 100%	19 (16)
Benefits^a,b^, *n* (%)	
Sick leave	12 (10)
Work assessment allowance	68 (56)
Disability pension	12(10)
Pension, work benefits, parental leave, or no benefits	33 (27)
Full return to work^d^	
Yes	27 (22)

a Data for 1 participant are missing.

b Participants could be on more than 1 benefit at the same time.

c Including participants with cancer, due to fewer than 5 participants.

d At the time of data collection.

e Occupations with 5 or more participants are named.

SD: standard deviation.

### Setting

This study is part of a larger research project named NOW WHAT – Rethinking Return to Work Services for Long-Term Sick Listed Employees. With Norway as its project setting, the NOW WHAT project aims to challenge how RTW services are designed, organized, and delivered as well as enable employees on long-term sick leave to resume their RTW processes. NOW WHAT includes several qualitative and quantitative sub-studies. The Norwegian social welfare system is funded by taxes, and compared with other countries, the sick-leave rates are high. Norwegian employees can receive up to a year of full-wage sick-leave benefits; the initial 16 days are paid by the employer, and the remaining time is paid by the social insurance system (Norwegian Labour and Welfare Administration [NAV]). After that, employees with reduced work ability (i.e., 50% or more) can apply for Work Assessment Allowance (WAA; Norwegian abbreviation AAP) for a maximum of 3 years before returning to work or apply for permanent disability benefits.

### Recruitment and participants

Participants were recruited in collaboration within a local geographical area of the NAV county office between 2018 and 2020. The eligibility criteria were employees receiving sick leave/WAA benefits for at least 50% of their working hours for a period between 6 and 18 months. Most participants had a full-time equivalent equal to 100% or above, as described in Table I.

The NAV directorate forwarded a short telephone text message (SMS) from the researchers to potential participants, inviting them to take part. A web link with information concerning the study was attached. By clicking on the link in the SMS, the potential participants entered a secure website, where informed consent to participate was obtained in addition to information including names, telephone numbers, and e-mail addresses.

This study included 53 females and 69 males whose ages ranged from 21 to 68 years, with a mean age of 47 years, as displayed in Table I. The participants had a variety of employment and benefit statuses. Work was classified according to the International Standard Industrial Classification of All Economic Activities. We used the International Classification of Primary Care to group information regarding the participants’ initial diagnoses for sick leave. Almost half of the participants reported diagnoses compatible with MSD (49%), approximately 21% with CMD, and 12% with CVD, while 18% were grouped as others, including CA (due to fewer than 5 participants) and neurological, respiratory, or endocrine/metabolic diagnoses.

### Data collection

The data were collected via telephone interviews from spring 2018 to spring 2020. Due to the relatively sensitive and specific topic of interest and overarching aim of gathering a broad spectrum of RTW barrier experiences, telephone interviews were considered a suitable method (35, 36). Before conducting the interviews, the principal investigator developed a 2-part interview guide. This guide was refined and tested in collaboration with master’s students who were trained in the interview procedure and participated in the data collection. The first part consisted of initial sociodemographic questions registered directly in a digital questionnaire. The second part consisted of 4 semi-structured open-ended questions, ranging from general to more specific questions, related to reasons for sick leave and self-perceived RTW barriers (e.g., “What obstacles or barriers do you experience that make it difficult for you to return to your work?”, “Can you think of any other barriers related to work/home or leisure activities”, and “Which of these obstacles or barriers do you perceive as the greatest in preventing your return to work? Please mention the three most important ones”). The interviewer’s role was to monitor and verify the interviewees’ responses. Three researchers and 8 master’s students participated in the data collection. All had health science and/or clinical backgrounds within the fields of occupational therapy, psychology, social work, nursing, or chiropractic. To ensure quality and transparency, we facilitated collaboration meetings with the individuals involved in the data collection (31, 33, 37). The interviews were audio-recorded with the participants’ consent and were transcribed verbatim.

### Data analysis

The transcripts were analysed using a combination of manifest deductive qualitative and quantitative content analysis approaches (38–42) with the software program NVivo 12 Pro (https://lumivero.com/products/nvivo/) and Microsoft Excel (Microsoft Corp, Redmond, WA, USA). First, the transcripts were read line by line and meaning units in alignment with the research question of self-perceived RTW barriers were identified. These meaning units were operationalized as words or sentences addressing RTW barriers. To stay close to the empirical data and limit interpretation of the text, the meaning units consisted primarily of the informants’ own words. Thereafter, we coded the meaning units according to the terminology of the International Classification of Functioning, Disability and Health (ICF) classification system (43). The ICF consists of 6 components: body functions (b), body structures (s), activity (a), participation (p), environmental factors (e), personal factors (P), and related categories. Prior to the data analysis, an ICF codebook was developed by the principal investigator that included all the ICF components and categories with descriptions (see Appendix S1 for an overview). The data analysis phase was conducted by 4 researchers and 1 master’s student and included training in the deductive content analysis coding procedure. All had health science and clinical backgrounds within the fields of occupational therapy, psychology, or nursing. Additionally, collaboration meetings were held to ensure the consistency of the coding frame across coders and to address any uncertainties regarding the coding of meaning units (38). The deductive content analysis process in line with the ICF framework is presented in Table II. To achieve greater structure in our material, identify patterns, and allow comparisons, we used numbers and conducted descriptive statistical analyses, such as frequency, percentage, and average number of meaning units per participant, as displayed in Table III.

**Table II:** Illustration of the deductive analysis process of self-perceived return to work barriers in accordance with the International Classification of Function, Disability, and Health (ICF)

ID	#	Interview transcript	Meaning unit description	ICF Component/code	ICF category/code
X	1	I: Are there other factors besides the main diagnosis that you see as the reason for not being able to return 100%? R: I guess I’d say that now it’s that you’re very tired; you can’t do what you did before	I guess I’d say that now it’s that you’re very tired; you can’t do what you did before	Body functions	b1 Mental functions – energy and drive functions
X	1	I: Yes, are there any other reasons you can think of? R: No, in terms of work. It’s the employer, but it has also been a struggle with social insurance services (NAV) in the beginning as well. With papers and wrong transmissions and employees who don’t know which papers they should have, and yes	But it has been a terrible struggle with social insurance services (NAV) in the beginning as well. With papers and wrong transmissions and employees who don’t know which papers they should have, and yes	Environmental factors	e5 Services, systems, and policies
X	1	I: Yes correct R: But otherwise, I’m healthy enough. I am healthy enough, as I stand as an ordinary person. It’s just that I can’t ... lift and be in heavy professions in the construction industry, so it will be too heavy	It’s just that I can’t … lift and be in heavy professions in the construction industry, so it will be too heavy	(a) Activity limitations	a8 Major life areas – work and employment

**Table III:** Self-perceived return-to-work barriers in accordance with the International Classification of Function, Disability, and Health (ICF) (*n *= 122)

ICF component	*n* *	Mean^a^ (SD)	%	Min, Max	95% CI
Body functions	552	4.5 (3.42)	28	0, 15	3.91–5.13
Environmental factors	414	3.4 (3.31)	21	0, 17	2.77–3.97
Activity limitations (a)	352	2.9 (2.91)	18	0, 14	2.38–3.41
Personal factors	323	2.6 (3.13)	17	0, 16	2.09–3.21
Participation restrictions (p)	151	1.2 (1.40)	8	0, 5	1.02–1.52
Body structures	146	1.2 (1.95)	8	0, 9	0.85–1.55
Total	1942	15.9 (9.26)	100	3, 57	14.25–17.57

* *n *= number of meaning units; no statistics are provided for meaning units with a frequency below 5 due to privacy concerns.

a Average per participant.

### Ethics

The project, which had gained approval from the Norwegian Centre for Research Data (NSD-57078), was presented to the Norwegian Regional Ethics Committee (reference no. 2018/2267) and received feedback that no approval was required, as it was assessed to be outside the committee’s mandate. Participation in the study was voluntary, and written informed consent was obtained prior to commencement. All data were treated confidentially, and personally identifiable information was anonymized.

## Results

We identified 1,942 meaning units with descriptions of self-perceived RTW barriers. The mean duration of the interviews was 15 min (min 12, max 42 min). As displayed in Table III, the frequency of coded barriers was highest for the body functions ICF component (*n* = 552, mean = 4.5 for each employee), followed by environmental factors (*n* = 414, mean = 3.4), activity limitations (*n* = 352, mean = 2.9), and personal factors (*n* = 323, mean = 2.6), and it was lowest for participation restrictions (*n* = 151, mean = 1.2) and body structures (*n* = 146, mean = 1.2).

### Body functions

Table IV provides an overview of the most reported barriers within the body functions component and the distribution of barriers for each category. Mental functions were the most frequent category (*n* = 229, mean = 1.9) (e.g., “I’ve got into a bad rhythm of sleeping very badly”), followed by sensory functions and pain (*n* = 157, mean = 1.3) (e.g., “It’s mostly the pains in my shoulders and neck and pelvis that I struggle with”), and neuromusculoskeletal and movement-related functions (*n* = 126, mean=1.0) (e.g., “I wasn’t able to lift my arm”).

**Table IV:** Self-perceived return-to-work barriers in accordance with the International Classification of Function, Disability, and Health (ICF) – Body functions component (*n *= 122)

ICF Component => Body functions	*n* *	Mean^a^ (SD)	%	Min, Max	95% CI
Mental functions (b1)	229	1.9 (2.41)	42	1, 11	1.44–2.30
Sensory functions and pain (b2)	157	1.3 (1.52)	29	1, 6	1.01–1.55
Neuro-musculoskeletal and movement-related functions (b7)	126	1.0 (1.74)	23	1, 9	0.72–1.34
Functions of the cardiovascular, haematological, immunological, and respiratory systems (b4)	20	0.2 (0.98)	4	1, 4	0.02–0.38
Functions of the digestive, metabolic, and endocrine systems (b5)	17	0.1 (4.02)	1	1, 11	–0.62–0.82
Genitourinary and reproductive functions (b6)	< 5				
Functions of the skin and related structures (b8)	< 5				
Voice and speech functions (b3)	< 5				
Total	552	4.5 (3.42)	100	0, 15	3.91–5.13

* *n *= number of meaning units; no statistics are provided for meaning units with a frequency below 5 due to privacy concerns.

a Average per participant.

Within the mental functions category, several participants reported RTW barriers concerning topics such as sleep (e.g., “It’s the fact that I don’t get to sleep for the most part”), tiredness (e.g., “I guess I’d say that now, when you’re very tired, you can’t do what you did before”), emotional functioning (e.g., “Thinking a lot, afraid of getting the cancer back again”) and cognitive functioning (e.g., “Eh ... the head can’t think; you get so tired that you can’t think”).

### Environmental factors

In the second most prevalent ICF component, environmental factors, the most frequent RTW barriers were related to the services, systems, and policies category (*n* = 257, mean = 2.1) and often concerned social security and the healthcare system, followed by support and relationship (*n* = 53, mean 0.4) (e.g., “Then there’s the fact that you always heard it; it wears you out psychologically that you’re only 50%, so somehow you shouldn’t be taken into consideration”) and attitudes (*n* = 52, mean = 0.4) (e.g., “What has been the biggest obstacle is that there is zero interest from the employer in adjusting in any way. Everything was rejected. They were not interested in doing it; they have no culture for facilitating”). Table V presents a detailed overview of the environmental factors component.

**Table V:** Self-perceived return-to-work barriers in accordance with the International Classification of Function, Disability, and Health (ICF) – Environmental factors component (*n *= 122)

ICF component => Environmental factors	*n* *	Mean^a^ (SD)	%	Min, Max	95% CI
Service, systems, and policies (e5)	257	2.1 (2.87)	63	1, 16	1.59–2.61
Support and relationship (e3)	53	0.4 (0.86)	13	1, 4	0.35–0.51
Attitudes (e4)	52	0.4 (1.63)	13	1, 6	0.14–0.72
Products and technology (e1)	34	0.3 (0.58)	8	1, 3	0.20–0.40
Natural environment and human-made changes to environment (e2)	18	0.1 (0.65)	4	1, 3	–0.02–0.22
Total	414	3.4 (3.31)	100	0, 17	2.77–3.97

* *n*=number of meaning units; no statistics are provided for meaning units with a frequency below 5 due to privacy concerns.

a Average per participant.

Within the services, systems, and policies category, topics concerned struggles with filling in forms (e.g., “The application form from NAV, for example: it was quite difficult, actually”), demanding processes (e.g., “And that was what I found most difficult about NAV, because when I wanted so much and I tried, every time I kind of collapsed again…. Then you had to apply again, and a new process and everything all over again”), and long waiting lists regarding treatment, assessment or medical operations (e.g., “Yes … it was because the doctors have such an incredibly long waiting time. So I had to wait over half a year to see a specialist, and the next time I went to the doctor, or to the specialist then, almost a year had passed. So what they tried last time was useless. Because there was such a long waiting time, I didn’t get any follow-up. And nothing”).

### Activity limitations

The third most often mentioned barrier component was activity limitations. The distribution of this component and its categories are presented in Table VI. The most frequent categories were major life areas (*n*=134, mean = 1.1) (e.g., “It’s just that I can’t … lift and be in the heavy professions in the construction industry; it would be too heavy”), followed by general tasks and demands (*n* = 68, mean = 0.6) (e.g., “I couldn’t focus on what I was supposed to do”), and interpersonal interactions and relationships (*n* = 50, mean = 0.4) (e.g., “The difficult thing is that I feel that they do not fully understand what is going on and they do not fully take into account the fact that I am actually in pain. And I struggle to do certain things at work. And then I think it will be very difficult to fully recover. They are unable to respect it because there is so much disease”).

**Table VI:** Self-perceived return-to-work barriers in accordance with the International Classification of Function, Disability, and Health (ICF) – Activity limitations component (*n *= 122)

ICF component => Activity limitations (a)	*n* *	Mean^a^ (SD)	%	Min, Max	95% CI
Major life areas (a8)	134	1.1 (1.76)	38	1, 10	0.78–1.42
General tasks and demands (a2)	68	0.6 (1.21)	19	1, 5	0.38–0.82
Interpersonal interactions and relationships (a7)	50	0.4 (1.93)	14	1, 7	0.05–0.75
Domestic life (a6)	37	0.3 (0.76)	11	1, 4	0.16–0.44
Mobility (a4)	34	0.3 (1.13)	10	1, 5	0.10–0.50
Community, social, and civic life (a9)	11	0.1 (0.74)	3	1, 3	–0.03–0.23
Learning and applying knowledge (a1)	10	0.1 (0.33)	3	1, 2	0.24–0.36
Self-care (a5)	7	0.1 (0.41)	2	1, 2	0.03–0.17
Communication (a3)	< 5				
Total	352	2.9 (2.91)	100	0, 14	2.38–3.41

* *n *= number of meaning units; no statistics are provided for meaning units with a frequency below 5 due to privacy concerns.

a Average per participant.

Within the major life areas category, many participants described their work as physically heavy work, and some mentioned that their bodies could no longer tolerate it (e.g., “No, that’s because it was too heavy. So I can’t do the same job; when it starts to get heavy, it takes a toll on my back. Then I can’t. It goes so fast that the back doesn’t want any more”).

### Personal factors

Personal factors, the fourth most reported RTW barrier component, and its categories are displayed in Table VII. The most reported barrier within this component was way of coping (*n* = 104, mean = 0.9) (e.g., “When you’re sort of trying your best to get well again, if I may say so … you also want to work so badly, so it’s no use wanting to work if you can’t work”), followed by physical shape (*n* = 63, mean = 0.5) (e.g., “No it’s my health”), and individual psychological resources (*n* = 36, mean = 0.3) (e.g., “That’s probably what I’m saying; the desire to take action is variable”).

**Table VII:** Self-perceived return-to-work barriers in accordance with the International Classification of Function, Disability, and Health (ICF) – Personal factors component (*n *= 122)

ICF component => Personal factors	*n* *	Mean^a^ (SD)	%	Min, Max	95% CI
Way of coping (P10)	104	0.9 (1.02)	42	1, 5	0.67–1.03
Physical shape (P12)	63	0.5 (1.25)	20	1, 6	0.28–0.72
Individual psychological resources (P14)	36	0.3 (1.33)	11	1, 6	0.06–0.54
Previous and present experiences (P7)	24	0.2 (1.07)	7	1, 4	0.01–0.39
Profession (P5)	20	0.2 (0.58)	6	1, 3	0.10–0.30
Personality traits (P13)	18	0.1 (0.41)	6	1, 2	0.03–0.17
Age (P3)	17	0.1 (0.67)	5	1, 3	–0.02–0.22
Habits (P9)	16	0.1 (0.26)	5	1, 2	0.05–0.15
Behavioural patterns (P11)	9	0.1 (0.49)	3	1, 2	0.01–0.19
Education (P4)	7	0.1 (0.96)	2	1, 3	–0.07–0.27
Lifestyle (P8)	6	0.05 (0)	2	1, 1	–
Upbringing, personal background (P6)	<5				
Sex (P1)	<5				
Race (P2)	<5				
Total	323	2.6 (3.13)	100	0, 16	2.09–3.21

* *n *= number of meaning units; no statistics are provided for meaning units with a frequency below 5 due to privacy concerns.

a Average per participant.

Within the way of coping category, participants described experiences related to topics such as lack of mastery experiences at work (e.g., “If my head could decide, I would have been at work a month after the operation, but I tried – let’s put it that way – and it didn’t go well”).

## DISCUSSION

The aim of this study was to explore self-perceived RTW barriers among employees with low educational levels on long-term sick leave. In this section, 4 main findings are discussed: (1) the wide spread of RTW barriers reported among all the ICF components, indicating complexity and comorbidity; (2) body functions, especially mental functions, dominated; (3) service, system, and policies represented 21% of all barriers and 63% of the environmental barriers, with the services of NAV and the healthcare system the most frequently mentioned barriers; and (4) activity limitations and personal factors were also frequently reported RTW barriers compared with the other ICF components.

### Broad spectrum of RTW barriers across all ICF components

Our main results displaying a multitude of barriers in all ICF components echo the results of 1 meta-review (9) and previously identified qualitative studies for long-term sick-listed employees across disorders (26–28). However, old age and being female were identified as common RTW barriers in the review by Cancelliere et al. (9) but were not frequently reported in our study. These differences may be due to differences in representativeness, which is a potential limitation of the present study, as discussed further in the limitations section. None of the previous qualitative studies specifically addressed employees with low educational levels or investigated the same time span of long-term sick-listed clients, thereby limiting direct comparison. However, the study by Dekkers-Sánchez et al. (28) predominantly involved participants with a low educational level in the Netherlands. This makes it more valid for comparison with our results. Specifically, the Dekkers-Sánchez study reported on 4 main barrier themes: health-related, personal, social, and work-related obstacles. This can be interpreted as support for and further confirms the relevance of our main findings regarding the commonly reported RTW barriers of body function, environmental factors, activity limitations, and personal factors. These findings underscore the need for stakeholders in the RTW process to assess a broader array of barriers beyond health-related functioning, such as environmental barriers, activity limitations, and personal factors, to better aid long-term sick-listed clients with low educational levels.

### High frequency of RTW barriers related to body and mental functions

The high frequency of reported RTW barriers related to body functions and, in particular, mental functions are comparable to previous research in this field (9, 10, 26–28). It is obvious that employees on long-term sick leave can experience mental health issues regardless of initial medical diagnoses. Nonetheless, our findings suggest that these are highly important barriers to addressing long-term sick-listed employees with low educational levels. Considering that highly educated individuals have been identified as overrepresented in RTW interventions (12), it might be especially important to examine ways to assist the group of sick-listed employees with low educational levels with the mental function barriers they experience and, in turn, provide better RTW services for this group.

### High level of environmental barriers, especially services, systems, and policy barriers

Another interesting finding is the high level of environmental barriers experienced, particularly those related to services, systems, and policies, such as NAV and the healthcare system. Obstacles related to these systems have been noted in other qualitative studies from the worker’s perspective (26–29), but they seem less investigated in reviews across disorders (9). Notably, Cancelliere et al. (9) did not report environmental RTW barriers related to services, systems, and policies as a common factor in their study. It is unclear whether this difference in findings can be viewed as contradictory to our results or whether service-related environmental factors were not explored in the previous study. Cancelliere et al. mentioned only work-related aspects in their description of environmental barriers.

Environmental factors are often considered in relation to the work arena in quantitative studies (9). A lack of focus on system influences was noted in a synthesis of international evidence on low back pain (44). Perhaps these services are designed in such a way that not everyone can make full use of them, and barriers are encountered when they are used. The term health service literacy may be relevant in this context. Here, it is emphasized that different actors must tune into service users’ needs and resources to increase the possibility of successful resource integration and mutual value (45). Our results suggest that these barriers require attention across disorders for long-term sick-listed people with low educational levels.

### Activity limitations and personal factors were relatively common RTW barriers

Although not as frequently reported as the above-mentioned RTW barriers in our study, both the major life areas category within the activity limitation component and the way of coping category within the personal factor component were relatively often reported compared with the other ICF categories. This implies that these categories are also important for long-term sick-listed people with low educational levels. Similar results were reported by Dekker-Sánchez et al. (28). Furthermore, these results can be interpreted in line with the existing theories of both the job demand–control (–support) model and the illness flexibility model (19, 20). Many of the participants in our study worked in what can be defined as high-strain jobs with high demands and low decision latitude. Recent research supports an association between low educational levels and high-strain jobs (18, 46). However, these models focus on the individual and their working context, while our findings regarding service, systems, and policies indicate a potential need to consider broader environmental aspects, including other system barriers. Furthermore, barriers related to activity limitations could be considered in relation to our findings of barriers related to navigating and interacting with the welfare system as well as accessing tailored RTW services.

### Implications for practice and future research

In line with previous research, we recommend that stakeholders in the RTW process take a broader perspective on RTW barriers for their clients regardless of the disorder, to better tailor their needs (9, 28, 29). Moreover, RTW interventions and RTW coordinators should aim to identify and break down barriers at multiple levels and in multiple arenas, especially mental health, system, and service aspects, in addition to activity limitations and personal factors for clients with low educational levels.

It remains to be investigated whether those with higher educational levels experience the same patterns of barriers. We will explore this perspective in more detail in a new study focusing on self-perceived RTW barriers for employees on long-term sick leave with high educational levels.

### Strengths/limitations

This study has several strengths, including a large sample size comprising a heterogeneous group of long-term sick-listed employees, which offered a rich pool of RTW experiences. The use of multiple interviewers and analyses with a theoretical framework using a deductive approach enhanced transparency and helped limit researcher bias.

Furthermore, conducting telephone interviews enabled us to reach a large geographical area while minimizing costs and the environmental load from travelling (47). We acknowledge that telephone interviews are relatively uncommon in qualitative research. However, telephone surveys are quite common and have been found to be appropriate for undertaking interviews on sensitive topics (36, 48, 49). Another potential limitation of telephone interviews is the lack of access to non-verbal communication, such as body language. However, the use of non-verbal information is not often included in the analysis of transcripts; moreover, the informants may have provided more honest responses because they were less likely to have been influenced by the interviewer’s presence on this sensitive topic (36).

The fact that this study employed a theory-driven analysis may have introduced confirmation bias, as the researchers may have been influenced to find results that support the theory (38). As the aim of the study was not to confirm or dispute the ICF theory, this potential bias was considered less likely for our purposes. We cannot rule out that using a different theory might have identified other RTW barriers for long-term sick-listed employees with low educational levels. We recommend that researchers in this field conduct comparative theory-driven analyses.

Moreover, in this study, we used numbers to display the count of meaning units across ICF codes, and these distributions need to be interpreted with caution (42). The findings of this study are not intended to be generalizable beyond the group studied; rather, it is a theory-driven exploratory descriptive study. Still, some of the participants were no longer on sick leave when the interviews were conducted, which may have contributed to information recall bias. However, this might also be a strength, as the participants who were no longer on sick leave may have overcome the barriers. Therefore, in hindsight, they might have been able to pinpoint the key barriers that hindered their RTW. Our sample also consisted of more male than female employees, and this may have limited our exploration of the RTW barriers associated more with female workers, such as work–family life imbalance (50). Additionally, it is important to consider that our sample predominantly consisted of individuals diagnosed with MSDs when interpreting our results. We did not aim for a fully representative sample in our study but wanted to provide in-depth knowledge regarding the lived experiences of RTW barriers to generate new hypotheses and stimulate further research in this field. Nonetheless, our results need to be replicated in other studies with representative samples drawn from the population of long-term sick-listed employees.

### Conclusion

Employees with low educational levels on long-term sick leave described a wide range of RTW barriers and combinations thereof. In addition to health-related functioning barriers, taking a broader perspective on RTW barriers, including environmental barriers, activity limitations, and personal factors, seems important to assess and increase insight into potential determinants of absence from work for this group.

## Supplementary Material


