# Supplementary material for: SELF-PERCEIVED BARRIERS TO RETURNING TO WORK AMONG EMPLOYEES WITH A LOW EDUCATIONAL LEVEL ON LONG-TERM SICK LEAVE: THE “NOW WHAT” LARGE-SCALE INTERVIEW STUDY
Source: J Rehabil Med. 2025 May 15;57:40604. doi: 10.2340/jrm.v57.40604 (PMC12105544; doi:10.2340/jrm.v57.40604)
Supplement: Supplementary file 1 [file JRM-57-40604-s1.pdf]

Supplementary material has been published as submitted. It has not been copyedited, or typeset by Journal of Rehabilitation Medicine

ICF Codebook with descriptions

Codes

Name	Description	Files	References
ICF			
FUNKSJON OG FUNKSJONSHEMMING /FUNCTION AND DISABILITY			
Aktivitet/Activity	«Aktiviteter er en persons utførelse av oppgaver og handlinger». «Aktivitetsbegrensninger er vanskeligheter en person kan ha ved å utføre aktiviteter». / "Activities are a person's execution of tasks and actions." "Activity limitations are difficulties a person may have in performing activities".		
A1 Læring og kunnskapsanvendelse/Learning and applying knowledge			
A2 Allmenne oppgaver og krav/general tasks and demand			
A3 Kommunikasjon/Communication			

Name	Description	Files	References
A4 Mobilitet/Mobility			
A5 Egenomsorg/Selfcare			
A6 Hjemmeliv/Domestic life			
A7 Mellommenneskelige interaksjoner og relasjoner/Interpersonal interaction and relationship			
A8 Viktige livsområder/Major life areas			
A9 Samfunnsliv og sosiale livsområder/Community social and civic life			
Deltagelse/Participation	«Deltagelse er å engasjere seg i en livssituasjon». «Deltagelsesinnskrenkninger er problemer en person kan oppleve ved deltagelse./ "Participation is engaging in a life situation. Participation restrictions are problems a person may experience with participation".		
P1 Læring og kunnskapsanvendelse/Learning and applying knowledge			
P2 Allmenne oppgaver og krav/General tasks and demand			

Name	Description	Files	References
P3 Kommunikasjon/Communication			
P4 Mobilitet/Mobility			
P5 Egenomsorg/Self Care			
P6 Hjemmeliv/Domestic life			
P7 Mellommenneskelige interaksjoner og relasjoner/ Interpersonal interaction and relationship			
P8 Viktige livsområder/ Major life areas			
P9 Samfunnsliv og sosiale livsområder/ Community social and civic life			
Kroppsfunksjoner (BODY FUNCTIONS)	«…er organsystemenes fysiologiske funksjoner, inklusive mentale Funksjoner» “Funksjonshemming er problemer ved kroppsfunksjoner og -strukturer, som ved feil eller tap av betydning”/”...are the physiological functions of body systems, including mental functions." "Disability is problems with body functions and structures, such as defects or loss of significance”		
B1 Mentale funksjoner/Mental functions			
B2 Sansefunksjoner og smerter/Sensory functions and pain			

Name	Description	Files	References
B3 Stemme og talefunksjoner/Voice and Speech functions			
B4 Kretsløp, blod, immun og respirasjonsfunksjoner/functions of the cardiovascular, heamatological, immunological and respiratory systems			
B5 Fordøyelse, stoffskifte og indresekretoriske funksjoner/ Functions of the digestive, metabolic and endocrine systems			
B6 Urinsystemets funksjoner, kjønnsfunksjoner og forplantning/ Genitourinary and reproductive functions			
B7 Nerve, muskel, skjelett og bevegelsesrelaterte funksjoner/ Neuromusculoskeletal and movement-related functions			
B8 Huden og tilhørende strukturers funksjoner/Functions of the skinn and related structures			

Name	Description	Files	References
Kroppsstrukturer (BODY STRUCTURES)	«…er anatomiske deler av kroppen, som organer, lemmer og deres enkeltdeler»/"...are anatomical parts of the body such as organs, limbs and their components".		
S1 Nervesystemets strukturer/Structures of the nervoussystem			
S2 Øye, øre og tilhørende strukturer/ The eye, ear and related structures			
S3 Stemme- og taledannende strukturer/Structures involved in voice and speache			
S4 Strukturer tilhørende kretsløpssystemet, blodsystemet, det immunologiske system og respirasjonssystemet/Structures of the cardiovascular, immunological and respiratory systems			
S5 Strukturer tilhørende fordøyelse, stoffskifte og endokrinsystemet/Structures related to the digestive, metabolic and endocrine system			

Name	Description	Files	References
S6 Strukturer med tilknytning til urinsystemet, kjønnsorganene og forplantningen/Structures related to the genitourinary and reproductive system			
S7 Bevegelsesapparatets strukturer/sStructures related to movement			
S8 Hud og tilhørende strukturer/Skin and related structures			
KONTEKSTUELLE FAKTORER /CONTEXTUAL FACTORS			
Miljø (ENVIRONMENTAL FACTORS)	Miljøfaktorer utgjør de fysiske, sosiale og holdningsmessige omgivelser hvor en person eksisterer og utfolder sitt liv. Disse faktorene er utenforliggende i forhold til individet, og kan ha en positiv eller negativ innvirkning på personens utfoldelse som samfunnsmedlem, på personens evne til å utføre handlinger eller oppgaver, eller på personens kroppsfunksjoner eller kroppsstrukturer./ Environmental factors constitute the physical, social, and attitudinal environments in which a person exists and lives their life. These factors are external to the individual and can have a positive or negative impact on the person's participation as a member of society, their ability to perform actions or tasks, or their body functions or structures.		
E1 Produkter og teknologi/Products and technology			

Name	Description	Files	References
E2 Natur og menneskeskapte miljøforandringer/Natural environment and human-made changes to environment			
E3 Støtte og sosialt nettverk/Support and relationships			
E4 Holdninger/Attitudes			
E5 Tjenester, systemer og strategier for tiltak/Services systems and policies			
Personlige faktorer (PERSONAL FACTORS)	Personlige faktorer er den spesielle bakgrunnen for en persons eksistens og livsutfoldelse, og omfatter særtrekk ved personen som ikke hører til noen helsetilstand eller noe helseforhold. Personlige faktorer klassifiseres ikke i ICF./ Personal factors are the unique background of a person's existence and life experiences, and include characteristics of the individual that are not part of any health condition or health-related issue. Personal factors are not classified in the ICF.		
P1 Kjønn/Gender			
P10 Mestringsmåter/Way of coping			
P11 Atferdsmønster/Behavioural pattern			
P12 Fysisk form/Physical shape			

Name	Description	Files	References
P13 Personlighetstrekk, karakter/Personality traits, character			
P14 Individuelle psykiske ressurser/Individual mental resources			
P2 Rase/Race			
P3 Alder/Age			
P4 Utdanning/Education			
P5 Yrke/Occupation			
P6 Oppdragelse, personlig bakgrunn og historie, sosial opprinnelse/Upbringing, personal background and history, social origin			
P7 Tidligere og nåværende livserfaring (tidligere og nåværende livshendelser og situasjoner)/Former and current life experiences, life events			
P8 Livsstil/lifestyle			
P9 Vaner/habits			
